# Effects of Topical Ozone Application on Outcomes after Accelerated Corneal Collagen Cross-linking: An Experimental Study

**DOI:** 10.18502/jovr.v15i3.7447

**Published:** 2020-07-29

**Authors:** Aysun Sanal Dogan, Canan Gurda, Sinan Caliskan, Evrim Onder, Figen Kaymaz, Elif Bilgic

**Affiliations:** ^1^Department of Ophthalmology, Saglik Bilimleri University, Diskapi Yildirim Beyazit Training and Research Hospital, Ankara, Turkey; ^2^Department of Pathology, Saglik Bilimleri University, Diskapi Yildirim Beyazit Training and Research Hospital, Ankara, Turkey; ^3^Department of Histology and Embryology, Hacettepe University, School of Medicine, Ankara, Turkey

**Keywords:** Ozone, Corneal Collagen Cross-linking, Corneal Confocal Microscopy, Corneal Oxygen, Experimental

## Abstract

**Purpose:**

Ozone is a trioxygen molecule that spontaneously degrades into oxygen and oxygen free radicals. This study was designed to assess the effects of topical ozone application on outcomes after corneal collagen cross-linking (CXL).

**Methods:**

Enucleated fresh cadaver yearling sheep eyes (*n* = 28) were divided into five groups: control (C, *n* = 6), sham (S, *n* = 6), ozone only (Z, *n* = 6), CXL only (X, *n* = 5), and Ozone + CXL (ZX, *n* = 5). In all groups, except C, the epithelial layer was removed. In group Z, 20 μg/mL liquid ozone was topically applied. In group X, CXL was performed in the accelerated pulse mode. In group ZX, both CXL and ozone were applied. Post-interventional oxygen levels were determined and corneal confocal microscopy and optical coherence tomography were performed. Corneas were evaluated using light and electron microscopy.

**Results:**

Pre-interventional central corneal thickness (CCT) was highest in the control group and considerably similar in the remaining groups (*P* = 0.006). Pre- and post-interventional CCT were significantly different in the ozonated groups (Z and ZX) (*P* = 0.028; *P* = 0.043). Demarcation line depths were similar in groups Z, X, and ZX (*P* = 0.343). Increased stromal tissue reflectivity was observed in groups Z, X, and ZX. Oxygen levels were higher in the ozonated groups (Z and ZX) (*P* = 0.006), and caspase activity was higher in the CXL groups (X and ZX) (*P* = 0.028) as compared to the other groups. Group ZX showed tighter, more regular, and parallel fibrils.

**Conclusion:**

Ozone increases corneal stromal oxygenation which can probably augment the effect of CXL. Future studies should investigate the safety and feasibility of ozone application during CXL.

##  INTRODUCTION

The cornea contains outer epithelial cell layer, middle stromal layer, and inner endothelial cell layer. The stromal layer comprises the major volume of the tissue and is composed of keratocytes and extracellular matrix (ECM) including collagen lamella. The shape and structure of the corneal stroma are primarily responsible for the transparency, strength, and contour of the cornea.^[1]^

Keratoconus (KC) is a non-inflammatory ectatic disorder of the cornea, characterized by progressively bulging and thinning in the central or paracentral area of the cornea.^[2]^ Ultrastructural changes of stromal matrix include increased corneal stromal protein degradation, decreased collagen lamella density, and disorganization of the stromal matrix, resulting in biochemical instability and weakening.^[3]^ Contemporary treatment has been undertaken with the advent of corneal collagen cross-linking (CXL), demonstrating promising results in terms of halting disease progression or even, to a limited extent, reversing the course of KC.^[4]^

The application of riboflavin and ultraviolet A during the CXL procedure results in the formation of oxygen radicals which crosslink in the adjacent collagen fibrils. This cross-linking increases the biomechanical strength by modifying the organization of the corneal lamellar structure, the diameter of the corneal stromal collagen, and the spacing between fibrils and proteoglycans.^[5]^ It has been shown that tissue oxygen is the key element for this reaction to occur.^[6]^

Within the last five years, a growing number of studies have been performed to facilitate the procedure without decreasing the efficacy. These studies have focused on the four basic variables of this procedure.

1 Delivery of riboflavin to cornea: Chemical de-epithelization, the “epi-on” (without de-epithelization) and intraoperative contact lens using techniques to decrease the operative complications and postoperative pain, and “iontophoresis technique” to increase efficacy have been described.^[7,8]^

2 Photosensitizing agent: Reportedly, studies have evaluated dosage modification and duration of riboflavin application; additionally, alternatives to this original photosensitizing agent have been investigated.^[9]^

3 Duration of exposure to UVA: The reaction is initiated by UVA. In 2014, “accelerated CXL” was described and aimed to shorten the duration by increasing the UVA irradiance, without changing the total energy.^[10]^

4 Oxygen: The photochemical reaction requires oxygen. Following the realization that environmental oxygen is rapidly depleted within seconds and rises to normal limits after 3 min, it was proposed that the “pulse CXL” method allows tissue reoxygenation.^[6,11,12]^

The effect of cross-linking on collagen was first investigated using the crystalline lens.^[13]^ The lens and lenticular collagen were the first subjects of these studies, which demonstrated the interaction among singlet oxygen, ozone, and applied riboflavin that resulted in free radical-induced cross-linking of the lenticular fibrils.^[14,15,16]^ These aforementioned studies put forward the possible use of ozone in CXL.

Ozone is an unstable trioxygen molecule. Its breakdown to oxygen gives rise to oxygen free radicals, which are highly reactive and powerful oxidizing agents.^[17]^ As a result, it serves as an oxygen supply. Therefore, by itself or in conjunction with riboflavin and UVA, it has the potential to augment the cross-linking effect. We hypothesized that ozone can be used as an adjuvant to the cross-linking reaction, as an oxygen generator, or as a cross-linking agent in the cornea. This study was designed to assess the effect of topical ozone application on the outcomes of the CXL procedure.

##  METHODS

### Study Design

Enucleated fresh cadaver yearling sheep eyes (*n* = 28) were obtained from a local slaughterhouse and the full experimental procedure was performed within 12 hours. The eyes were divided into five groups: control (C, *n* = 6), sham (S, *n* = 6), ozone only (Z, *n* = 6), CXL only (X, *n* = 5), and Ozone + CXL (ZX, *n* = 5).

During the experiment, the eyes were handled from the equator using gauze sponges. Group C was not touched or handled, and the epithelial layer was mechanically removed for all other groups. In group S, only the epithelial layer was mechanically removed. In group Z, 20 μg/mL ozonated water was topically applied to the de-epithelized cornea. In group X, CXL was performed using a total energy of 5,4 J/cm${}^{2}$ in the accelerated pulse mode.^[11]^ In group ZX, ozone was first applied, followed by CXL treatment. Pre- and post-interventional anterior segment optical coherence tomography (AS-OCT) and corneal confocal microscopy (CCM) were performed. Immediate post-interventional corneal stromal oxygen levels were measured. After the procedure, each cornea was dissected from the globe and specimens were obtained to perform light and electron microscopic evaluations.

### Ozone Application

Liquid ozone (20 μm/mL) was obtained using an ozone generator (Refresh, Refreshozon Medical, Ankara, Turkey) in distilled water in a separate room. Two milliliters of the ozonated water was applied to the cornea for 2 min using a silica glass apparatus, with a corneal 8 mm aperture.

### Cross-linking

After removal of the central 8mm portion of the epithelium using a No. 15 knife, 0.1% riboflavin (vitamin B2) with hydroxypropyl methylcellulose (VibeXRapid, Avedro) was applied drop-wise to the cornea at 2 min intervals for 10 min to achieve corneal penetration. Anterior segment diffusion was controlled using handheld biomicroscope. A UVA (370 nm) generator designed for corneal cross-linking was used (KXL System, Avedro Inc., Waltham, MS, USA). First, guiding lights were set to focus the UVA onto the cornea perfectly. The parameters were set as power: 15 mW/cm${}^{2}$, a total energy of 5.4 J/cm${}^{2}$ for each eye, with a pulse of 1.5-sec on and 1.5-sec off mode.

### Oxygen Measurement

Post-interventional oxygen measurements were performed at room temperature (21ºC). The operator was masked to the groups. An oxygen sensor microprobe (PreSens, Regensburg, Germany), with a tapering end < 50 μm in diameter, was placed from the epithelized cornea, passing 2 mm into the de-epithelized cornea, with a parallel angle at the half-thickness of the cornea under the biomicroscope. The mean of three consecutive measurements was used for statistical analyses.

### Anterior Segment Optical Coherence Tomography (AS-OCT) 

Corneas were imaged using optical coherence tomography (OCT, RTVue-XR, Optovue Inc., Fremont, CA). The non-contact anterior segment attachment lens (CAM-L), corneal line mode, and automated image analysis system were used. The sections were set to the central cornea. In particular, central corneal thickness (CCT) was measured in micrometers and the depth of demarcation lines, which are the results of different reflectivity, were investigated. The reflective line between the cross-linked and untreated areas was determined and marked.

### Corneal Confocal Microscopy (CCM)

Laser scanning CCM (HRT III-RCM, Heidelberg Engineering, Dossenheim, Germany) was used to evaluate the cellular structures of the corneas. A transparent ophthalmic gel (Viscotears Ophthalmic Gel, Alcon) was filled into the confocal cap attached to the objective lens and on its external surface. The eyes were handled with a sponge from the equator, providing the central corneal touch to the cap. The captured images were analyzed by a clinician (ASD) experienced in CCM.

### Light Microscopy

Caspase-3 staining was performed to detect keratocyte apoptosis. Paraffin-embedded tissue blocks were sectioned into 4-5µm thick slides. The slides were deparaffinized, rehydrated, and washed in phosphate-buffered saline (PBS). After treatment with 3% hydrogen peroxide in aqueous solution, the sections were blocked with PBS-6% non-fat dry milk for 1 hour at room temperature. The slides were then incubated at 4ºC overnight with the primary antibody for cleaved caspase-3 (CPP32) (Thermo Scientific). After washing with PBS, the slides were treated with a solution of diaminobenzidine (DAB). Finally, a counterstain with hematoxylin was performed and the slides were allowed to dry. The immunohistochemical (IHC) evaluation was performed to determine the nuclei of stromal cells, and positivity was scored as percentages. Caspase activity was evaluated in stromal cells. In each section, the area with the highest density of nuclear positivity was selected and 100 cells were counted. The ratio of positively stained nuclei to 100 (the percentage value) was accepted as the caspase activity for each given case.

### Electron Microscopy

Tissue samples were carefully dissected and 1 mm${}^{3}$-sized samples were fixed overnight in 2.5% glutaraldehyde in PBS. Next, the tissue samples were washed with PBS and fixed with 1% osmium tetroxide solution. After washing with PBS, the tissue samples were dehydrated with a graded alcohol series. An Araldite/Epon812 mixture (Cat no.: 13940, EMS, Hatfield, PA, USA) was used to embed the propylene-oxide-treated tissue. The tissue blocks obtained were maintained at 60ºC for two days to complete polymerization. Thin sections were obtained using the Leica Ultracut R, and contrast double-stained using uranyl acetate and lead citrate (Leica EM AC20). Next, examinations were performed by using transmission electron microscope (TEM; JEM 1400, Jeol, Japan) with an attached digital CCD camera (Gatan Inc., Pleasanton, CA, USA). To compare the differences among the groups, we obtained four blocks from each group, and then examined four sections from each block. Finally, collagen fibril diameters and ECM distances from four non-overlapping areas were calculated at the magnification of 100k.

##  RESULTS

**Figure 1:**
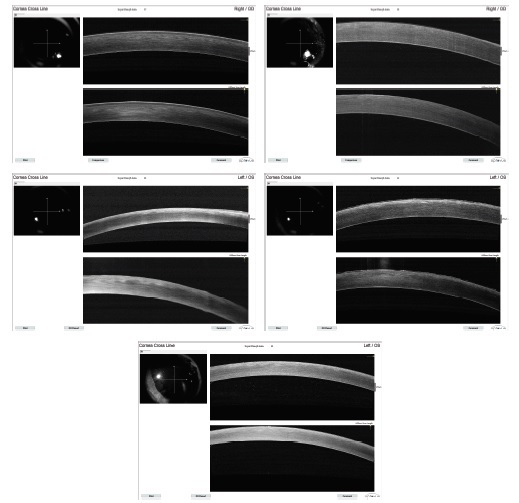
Optical Coherence Tomography corneal images of the groups control (a), sham (b), ozone (c), cross-linking (d), ozone-cross-linking (e), respectively. Hyperreflectivity is increased in anterior stroma in groups X and Z, but generalized in ZX.

Tissue oxygen levels were higher in the ozonated groups (Z and ZX) than in the other groups (*P* = 0.006, Kruskal–Wallis test). Pre-interventional CCT measures were higher in the control group (C) as compared to the other groups (*P* = 0.006); no significant difference, however, was found among the remaining groups in the baseline CCT. Post-interventional CCT differed significantly among the groups (*P* = 0.001). Compared to the baseline values, the post-interventional CCT decreased significantly in groups Z and ZX (Group Z: *P* = 0.028, Group X: *P* = 0.768, Group ZX: *P* = 0.043; Table 1). Similar demarcation lines were observed in the interventional groups, although group ZX showed a generalized increase in corneal reflectivity (Figure 1).

**Table 1:** Between and within groups comparison of the examined parameters.


	**Group C**	**Group S**	**Group Z**	**Group X**	**Group ZX**	**p${}^{a}$**
Caspase activity, %, median (range)	2.5 (1–4)	2 (1–3)	2.5(1–5)	**3 (3–5)**	**4 (3–6)**	0.028*
Oxygen saturation, %, median (range)	18.7 (14.5–22.2)	16.7 (11.9–22.3)	**21.7 (20.4–26.0)**	16.4 (16.0–18.7)	**22.2 (21.3–23.3)**	0.006**
CCT, preop, mcm, median (range)	**673.5 (664–768)**	587.5 (545–639)	637 (528–653)	609 (571–655)	609 (545–652)	0.006***
CCT, postop, mcm, median (range)	673.5 (664–768)	587.5 (545–639)	569 (506–635)	622 (555–657)	541 (532–558)	0.001****
Demarcation, mcm, median (range)		203.5 (185–257)	190 (185–245)	323 (150–360)	0.343
Group C: control, Group S: sham, Group Z: ozonated only, Group X: cross-linked only, Group ZX: ozonated and crossl-linked, CCT: central corneal thickness, a: Kruskal–Wallis test **Between groups comparisons:** *Group C vs ZX, *p* = 0.0025; Group S vs X, *p* = 0.021; Group S vs ZX, *p* = 0.009; Mann–Whitney U-test for each **Group C vs Z, *p* = 0.037; Group C vs ZX, *p* = 0.018; Group S vs Z, *p* = 0.025; Group S vs ZX, *p* = 0.028; Group Z vs X, *p* = 0.006; Group X vs ZX, *p* = 0.009, Mann–Whitney U-test for each ***Group C vs S, *p* = 0.004; Group C vs Z, *p* = 0.004; Group C vs X, *p* = 0.006; Group C vs ZX, *p* = 0.006; Mann–Whitney U-test for each ****Group C vs S, *p* = 0.004; Group C vs Z, *p* = 0.004; Group C vs X, *p* = 0.006; Group C vs ZX, *p* = 0.006; Group S vs ZX, *p* = 0.018; Group X vs ZX, *p* = 0.028; Mann–Whitney U-test for each **Pre- vs post-operative pachymetry (CCT) comparison within study groups: **Group Z: wilcoxon signed rank test, *p* = 0.028; Group X: wilcoxon signed rank test, *p* = 0.768;****Group ZX: wilcoxon signed rank test, *p* = 0.043			

**Table 2:** Collagen fibril diameter and distance between fibrils regarding the groups which were measured by TEM.


**Groups**	**Collagen diameter in nm, median (range)**	**Distance between collagens in nm, median (range)**
N (No intervention [C + S])	24,6 (14.1–49.4)	25.2 (9.1–61.6)
Z	23.7 (16.7–35.0)	24.3 (11.5–44.6)
X	23.1 (15.6–34.9)	27.7 (16.2–50.4)
ZX	23.2 (16.4–32.3)	18.3 (9.1–40.9)
TEM, transmission electron microscope; N, no intervention (C: control and S: sham); Group C, control; Group S, sham; Group Z, ozonated only; Group X, cross-linked only; Group ZX, ozonated and cross-linked		

**Table 3:** Between groups comparison of collagen fibril diameter and distance between fibrils.


	**Collagen fibril diameter (** ***p*** **-value)${}^{a}$**	**Distance between collagen fibrils (** ***p*** **-value)${}^{a}$**
Group N vs Z	< 0.001	0.289
Group N vs X	< 0.001	< 0.001
Group N vs ZX	< 0.001	< 0.001
Group Z vs X	0.029	< 0.001
Group Z vs ZX	0.200	< 0.001
Group X vs ZX	0.337	< 0.001
N, no intervention (control and sham); Group Z, ozonated only; Group X, cross-linked only; Group ZX, ozonated and cross-linked; a, Mann–Whitney U-test		

**Figure 2:**
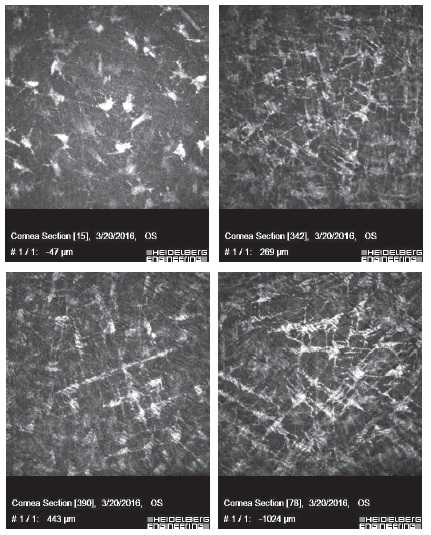
Corneal confocal images of groups sham (a), ozone (b), cross-linking (c), ozone-cross-linking (d), respectively. The keratocytes in stromal level shows straight extensions, which are signs of activity in all groups except control, which are exaggerated in ZX group.

**Figure 3:**
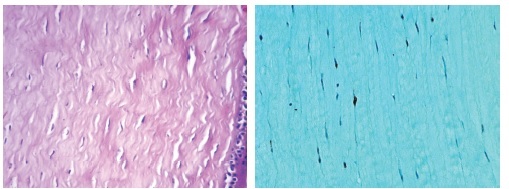
(a) A section of cornea from control group (HE X 400). (b) Positively stained nuclei in a section from ZX group (Caspase X 400).

**Figure 4:**
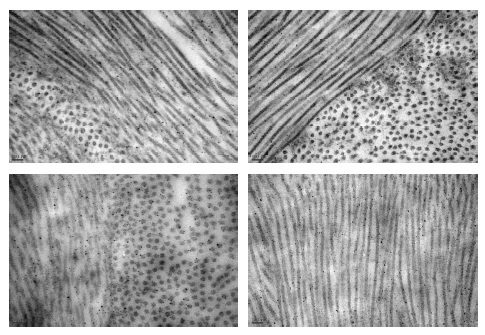
Images taken from transmissional electron microscopy of the corneas from groups (x100K); control (a), ozone (b), cross-linking (c), ozone-cross-linking (d), respectively. Collagen fibers and interlamellar spaces were examined by electron microscopy.

CCM demonstrated corneal stromal hyper-reflectivity (group Z, X, and ZX) in all interventional groups; however, the hyper-reflectivity was marked and present in all layers of the stroma in group ZX (Figure 2).

The cross-linked groups (groups X and ZX) showed higher caspase activity, with significant differences observed in caspase-3 staining between groups C and ZX, between groups S and X, and between groups S and ZX (*P* = 0.025, *P* = 0.021, *P* = 0.009, respectively) (Table 1, Figure 3).

The TEM findings and images revealed similar collagen fibril organization and undulation in groups C and S. The fibrils were compact and randomly distributed in group Z, oriented in a parallel pattern in group X, more compact and tightly arranged in group ZX (Figure 4).

The collagen fibril diameters were higher in the non-interventional groups (groups C and S). In the interventional groups, the collagen fibrils were thicker in group Z than in the other groups (Tables 2 and 3). The distance between collagen fibrils was lowest in group ZX and highest in group X (Tables 2 and 3).

##  DISCUSSION

Oxygen radicals are present in normal tissues and cells. However, their effect is dose-dependent with a possible destructive effect on the cell wall structure and genetic material.^[18]^ Ozone (O${}_{3}$) is normally found in the stratosphere and is used for conventional drinking water production at a concentration of 1–3 µl/mL.^[19]^ It is a highly reactive, unstable gas that spontaneously degrades to oxygen radicals and acts as an oxygen provider.^[17]^ There are three main types of molecular ozone reactions: (1) Electron transfer reactions, resulting in free oxygen radicals; (2) Oxygen-atom transfer reactions mainly occurring in inorganic materials; (3) Ozone addition reactions, which are the primary reactions occurring in organic compounds, resulting in double bond formation.^[20]^ Our study revealed that oxygen levels in the corneal stroma were higher in ozonated groups, which supports our primary goal, the oxygen-providing effect.

Several studies have evaluated the effects of ozone molecules on the eye. An experimental study revealed that ozone resulted in a decrease in goblet cell density and an increase in inflammatory cytokines on the ocular surface.^[21]^ Our study showed that corneal thickness was decreased in the ozonated groups (groups Z and ZX). This observation can be explained by the use of liquid ozone which induced this decrease in thickness owing to its osmotic effect. To minimize this effect, we preferred the maximum possible concentration and the lowest exposure time. Wu et al^[22]^ have shown that ozone decomposes rapidly to diatomic oxygen and has a short half-life of 1–10 min in water.^[22]^ We applied ozone through a silica tube with an 8 mm aperture to limit the contact time within this range and prevent corneal edema.

Kamaew et al have shown that the stromal oxygen concentrations decrease within the first 15 sec of UVA application and then rise to normal levels 3 min after cessation of UVA.^[12]^ Oxygen is necessary for the photochemical reaction to occur during the CXL procedure.^[6]^ In our study, the microprobe measurements showed higher corneal stromal oxygen levels in ozonated groups (groups Z and ZX), suggesting penetration of the topically applied ozone to the stroma. This assumption, however, needs further refinement.

The standard CXL procedures lasts up to 60 min and a pulsed accelerated protocol has been developed to shorten the treatment duration without losing the therapeutic efficacy.^[11,23]^ Therefore, we aimed to augment the CXL effect in the pulsed accelerated protocol and, in the case of encouraging findings, implement this modification to clinical settings.

Early demarcation was previously described in an experimental study by Zhu et al; they postulated that the early apoptosis of keratocytes could induce increased light scattering and demarcation between the affected and unaffected stroma.^[24]^ We detected that demarcation lines in groups Z and ZX were similar to those in group X, indicating that the ozone itself triggers a chemical reaction similar to that observed in CXL. Post-interventional CCM findings demonstrated that increased stromal reflectivity and matrix striation were prominent in the anterior part in groups Z and X, whereas these changes were present throughout the stromal layer in group ZX. These results confirmed that ozone can potentially augment the cross-linking effect of the classical CXL procedure.

Another study has indicated anterior stromal keratocyte apoptosis following CXL.^[25]^ We detected apoptosis with a higher caspase activity in cross-linking groups (groups X and ZX), which demonstrates the efficacy of CXL.

CXL allows the reorganization of the corneal lamellar tissue by modifying the stromal collagen diameter and distance between collagen fibrils and proteoglycans.^[26]^ Clinical studies have revealed a mean collagen diameter of 30.8 nm and 32.2 nm in individuals younger and older than 65 years, respectively.^[27]^ It reveals that a natural cross-linking occurs non-enzymatically via free radicals with increasing age.^[28]^

Furthermore, we examined the effect of these procedures on collagen fibrils by utilizing TEM. The obtained images supported our hypothesis regarding the distribution pattern of collagen fibrils. In the ozonated group (group Z), the fibrils were denser than the controls and demonstrated a random distribution pattern. In the CXL group (group X), the fibrils were markedly condensed and presented an undulated pattern; in the ZX group, the undulation was less prominent and the distribution was more uniform and dense. These findings showed that the ozone application had an augmenting effect on the CXL procedure. Conversely, our measurements of fibril diameter and interfibrillar distance were not in accordance with our hypothesis. The fibril diameters of the study groups were similar but smaller than those of the control and sham groups, and interfibrillar distance was decreased from group X to group Z to group ZX in descending order (Table 2). The small number of eyes in the groups, the random selection of four ultrastructural counting areas, and manual counting could be the principal explanations for this contradicting finding.^[29]^

Ozone is an inert, cheap, and easily available molecule that induces covalent chemical bonds resulting in cross-linking. Our study is the first to evaluate the effects of this molecule on the results of the CXL procedure. Our findings in cadaveric eyes can be regarded as preliminary to potential clinical applications. However, further animal studies are warranted to determine the most appropriate concentration, dosage, and duration of application of ozone as well as safety issues. These studies should evaluate the osmotic effects of the gaseous ozone form and the biomechanical properties of the treated corneas.

##  Financial Support and Sponsorship

This work was funded by the Scientific and Technological Research Council of Turkey (TUBITAK) [grant numbers: 115S862].

##  Conflicts of Interest

There are no conflicts of interest.
